# The Elderly Nursing Core Set and the cognition of Portuguese older adults: a cross-sectional study

**DOI:** 10.1186/s12912-021-00623-1

**Published:** 2021-06-23

**Authors:** César Fonseca, Lara Guedes de Pinho, Manuel José Lopes, Maria do Céu Marques, José Garcia-Alonso

**Affiliations:** 1 Escola Superior de Enfermagem São João de Deus, Universidade de Évora, Largo do Sr. da Pobreza, 2B, 7000 − 811 Évora, Portugal; 2 Comprehensive Health Research Centre (CHRC), Évora, Portugal; 3 University of Extremadura, Cáceres, Spain

**Keywords:** Older adult, Cognition, Functioning, Nursing home, Multimorbidity, Geriatric nursing

## Abstract

**Background:**

The aging population and its associated health needs require specific nursing care. The aim of this study was to draw an epidemiological profile of Portuguese elderly adults attending in residential homes and day centers and to evaluate the association between the functioning and cognition of these older adults and their sociodemographic characteristics and presence of multimorbidity.

**Methods:**

This was a cross-sectional study of 613 older adults. Functioning was assessed using the Elderly Nursing Core Set, and cognition was assessed using the Mini Mental State Examination. Descriptive and inferential analyses were performed.

**Results:**

The mean age was 85.73 years; the majority of the participants were female (69.3 %), widowed (67.0 %) and over 85 years old (60.4 %). A total of 68.2 % of the sample presented multimorbidity. A total of 54.5 % had cognitive impairment, and the average functional profile was classified as “moderate difficulty”. Institutionalized older adults had more diseases than those who attended the day center. Women, those who were illiterate, those who were institutionalized and older adults who had diseases of the nervous system had a worse functional profile and greater cognitive impairment. Those with multimorbidity had a worse functional profile, and those without a spouse had greater cognitive impairment.

**Conclusions:**

Given the functional and cognitive profile of older adults, it is necessary to adopt care practices focused on the rehabilitation/maintenance of self-care and affective relationships. This care must be provided by highly qualified professionals. Therefore, it is necessary to increase the ratio of nurses per older adult in these institutions.

## Background

Both physical and cognitive limitations can lead to a loss of independence in performing activities of daily living, making an individual dependent on others [1]. The functioning of older adults may be influenced by the sociodemographic, cultural and environmental context [2–4]. When limitations cannot be overcome with help from others, mechanical assistance or changes in the environment, the consequences can compromise the individual’s ability to perform activities of daily living and participate in society [5].

The decline in functioning, including cognitive function, and the prevalence of chronic diseases tend to worsen with age and is particularly evident starting at 80 years of age [6–10].

Cognitive decline is rarely evaluated, and such evaluation is especially important in individuals with multimorbidity because many people with cognitive decline also have limitations in performing activities of daily living, which hinder or prevent their self-management of diseases [11].

A recent study concluded that understanding the heterogeneity in chronic pathologies, functional limitations, geriatric syndromes and causes of death in people with cognitive impairment can contribute to adequate care management and resource allocation [11]. These factors must be considered in care management at institutions for older adults, such as in residential homes.

In Portugal, institutions for older adults comprise essentially three main modalities (day centers, home care services and residential homes). These institutions are the responsibility of the social security sector [12], which differs from other countries that have adopted the concept of “nursing homes” that provide 24-hour health care services [13]. In nursing homes, older adults with functional limitations, whether physical or mental, who require care and supervision are monitored by nurses. In Portugal, many residential homes offer nursing care for only a few hours a day, and the number of hours that care is provided is not guaranteed or regulated according to the needs of the people. The current legislation defines that only one nurse is required for every 20 to 40 residents, depending on the degree of dependence [14], and the method used to evaluate the degree of dependence has not been defined.

In view of these factors, we consider it necessary to identify the sociodemographic and pathological characteristics of older adults attending institutions for older adults in Portugal. In addition, it is essential to expand the scientific knowledge of the factors associated with functional and cognitive limitations and multimorbidity. Thus, this study had the following aims:

1. To analyze the sociodemographic and pathological characteristics of older Portuguese adults attending in residential homes and day centers;
2. To evaluate the association between the functioning and cognition of older adults and their sociodemographic and pathological characteristics.

## Methods

### Study design and participants

This was a cross-sectional, descriptive and correlational study of a quantitative nature.

The sample was composed of older adults attending 18 institutions from northern to southern Portugal. Nonprobabilistic convenience sampling was used for sample selection. All older adults from the selected institutions were considered for participation.

The sample was composed of 613 older adults who attended either a day center (*n* = 507) or a residential institution (*n* = 106). The inclusion criteria were as follows: being 65 years of age or older and having the ability to sign informed consent or having a legal representative to do so. Participants who were excluded consisted of those under the age of 65 and those who were unable to give informed consent, as the legal representative was not present at the time the data were collected.

### Data collection instruments

In addition to the questionnaires administered, the following data were also collected: age, sex, marital status and education level. Information on diagnostic categories was also collected in the clinical process.

#### Mini Mental State Examination (MMSE)

The MMSE evaluates cognitive function and was developed by Folstein et al. (1975). The MMSE consists of six groups of questions that evaluate temporal and spatial orientation, recall, attention and calculation, repetition, language and constructive capacity [15]. Better scores indicate better cognitive ability[15]. The MMSE was adapted for the Portuguese population by Guerreiro and colleagues[16, 17].

#### Elderly Nursing Core Set (ENCS)

The ENCS was developed by Fonseca and collaborators, and it is used to assess the functioning of older adults[3]. The ENCS consists of 25 questions based on the International Classification of Functioning, Disability and Health (ICF) and is scored on a Likert scale from 1 to 5 points. The resulting scores yield a functional profile, as follows: (1) No disability: 0–4 %; (2) Mild disability: 5–24 %; (3) Moderate disability: 25–49 %; (4) Severe disability: 50–95 %; and (5) Complete disability: 96–100 %. The study that evaluated the psychometric characteristics of this tool showed a Cronbach’s alpha of 0.963[3]. The ENCS consists of four domains that are subdivided into various ICF codes: self-care, learning and mental functions, communication and social relationships[3]. The higher the score, the worse the functional profile of the individual[6].

### Data collection procedures

After positive feedback from the ethics committee approval, permission to participate in the study was requested from the management of each of the institutions.

The researchers who collected the data received prior training on how to conduct the interviews. Data were collected between July 2019 and February 2020 at the institutions in the day center or residential home setting. Interviews were conducted to collect data from users and/or their family and/or health professionals at the institutions. Data on the total number of valid interviews were entered into the Multidimensional Integrated Assessment Platform for Elderly (MIAPe) platform[18]. The researchers recorded the responses on the MIAPE platform, assigning a code to each participant so that they would not be identified. The data were then exported from the platform to an Excel file and from there to SPSS so that they could be analyzed.

### Ethical considerations

 Authorization was obtained from the Ethics Committee for Scientific Research in the Areas of Human Health and Welfare of the University of Évora under reference number 19,013.

All methods were performed in accordance with the Declaration of Helsinki of 1964 and its subsequent amendments[19]. Participation in the study was explained to the participants, and informed consent was obtained from the participant or a legal representative if the participant was not able to provide it.

### Data analysis

The data were analyzed using IBM SPSS Statistics version 24 for Windows. The sociodemographic variables were analyzed using descriptive statistics. The normality of the data was tested using the Kolmogorov-Smirnov test, and in the absence of a normal distribution, parametric tests were used given the sample size [20]. Thus, Pearson’s correlation was used to analyze the differences in age and the ENCS and MMSE scores. The independent *t*-test for independent samples was used to analyze differences in the ENCS or the MMSE scores, some sociodemographic characteristics and multimorbidity.

## Results

### Sample characteristics

The sample consisted of 613 older adults with a mean age of 85.73 years (± 6.890) and an age range from 65 to 100. Most were female (69.3 %), widowed (67.0 %) and over 85 years old (60.4 %). Regarding the nosological diagnosis, data could be obtained for only 572 participants, of whom 68.2 % had two or more diagnoses (multimorbidity) and only 0.5 % did not have any pathology (Table 1).

**Table 1:** Sample characteristics (*N* = 613)

	n (%)
**Sex**	
Female	425 (69.3)
Male	188 (30.7)
**Age**	
65-74 years	40 (6.5)
75-84 years	203 (33.1)
> 85 years	370 (60.4)
**Marital Status**	
Single	67 (10.9)
Married	109 (17.8)
Widower	411 (67.0)
Divorced	26 (4.2)
**Education**	
Illiterate	195 (31.8)
Did not go to school but knows how to read and write	29 (4.7)
Attended school but not higher education	369 (60.2)
Higher education	20 (3.3)
**Residence**	
Day center	106 (17.3)
Residential home	507 (82.7)
**Multimorbidity (*n*= 572)**	
Yes	390 (68.2)
No	182 (31.8)
No data	41
**Cognitive deterioration (MMSE)**	
Yes	334 (54.5)
No	279 (45.5)
	*Mean (DP)*
**Functional profile (ENCS)**	2.34 (1.132)
Self-care	2.72 (1.259)
Learning and mental functions	2.41 (1.127)
Communication	1.89 (1.268)
Social relationships	1.84 (0.597)

Regarding the functional and cognitive assessments, 54.5 % had cognitive impairment, and the sample as a whole had an average overall functional profile of “moderate disability” (2.34 (± 1.132)), with the worst results in the self-care domain, followed by the learning and mental function, communication and social relationship domains.

**Fig. 1:**
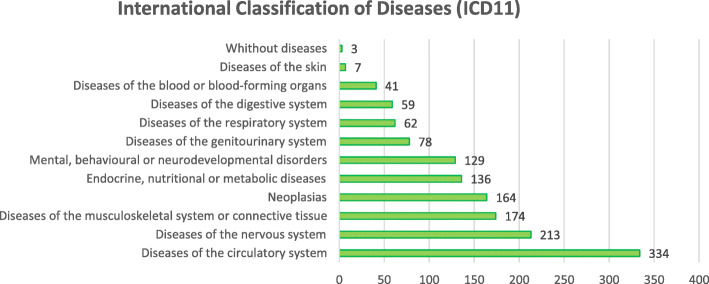
Percentage of participants by diagnostic categories (*n* = 572)

The most prevalent diagnostic category area was circulatory diseases, followed by diseases of the nervous system, including dementia; musculoskeletal system diseases; neoplasms; endocrine, nutritional and metabolic diseases; and mental and behavioral disorders (Table 2; Fig. 1).

**Table 2:** Percentage of participants by diagnostic category

Diagnostic category	N (%)
Diseases of the circulatory system	334 (54.5)
Diseases of the nervous system	213 (34.7)
Diseases of the musculoskeletal system or connective tissue	174 (28.4)
Neoplasias	164 (26.8)
Endocrine, nutritional or metabolic diseases	136 (22.2)
Mental, behavioral or neurodevelopmental disorders	129 (21.0)
Diseases of the genitourinary system	78 (12.7)
Diseases of the respiratory system	62 (10.1)
Diseases of the digestive system	59 (9.6)
Diseases of the blood or blood-forming organs	41 (6.70)
Diseases of the skin	7 (1.1)
Without diseases	3 (0.5)

**Fig. 2:**
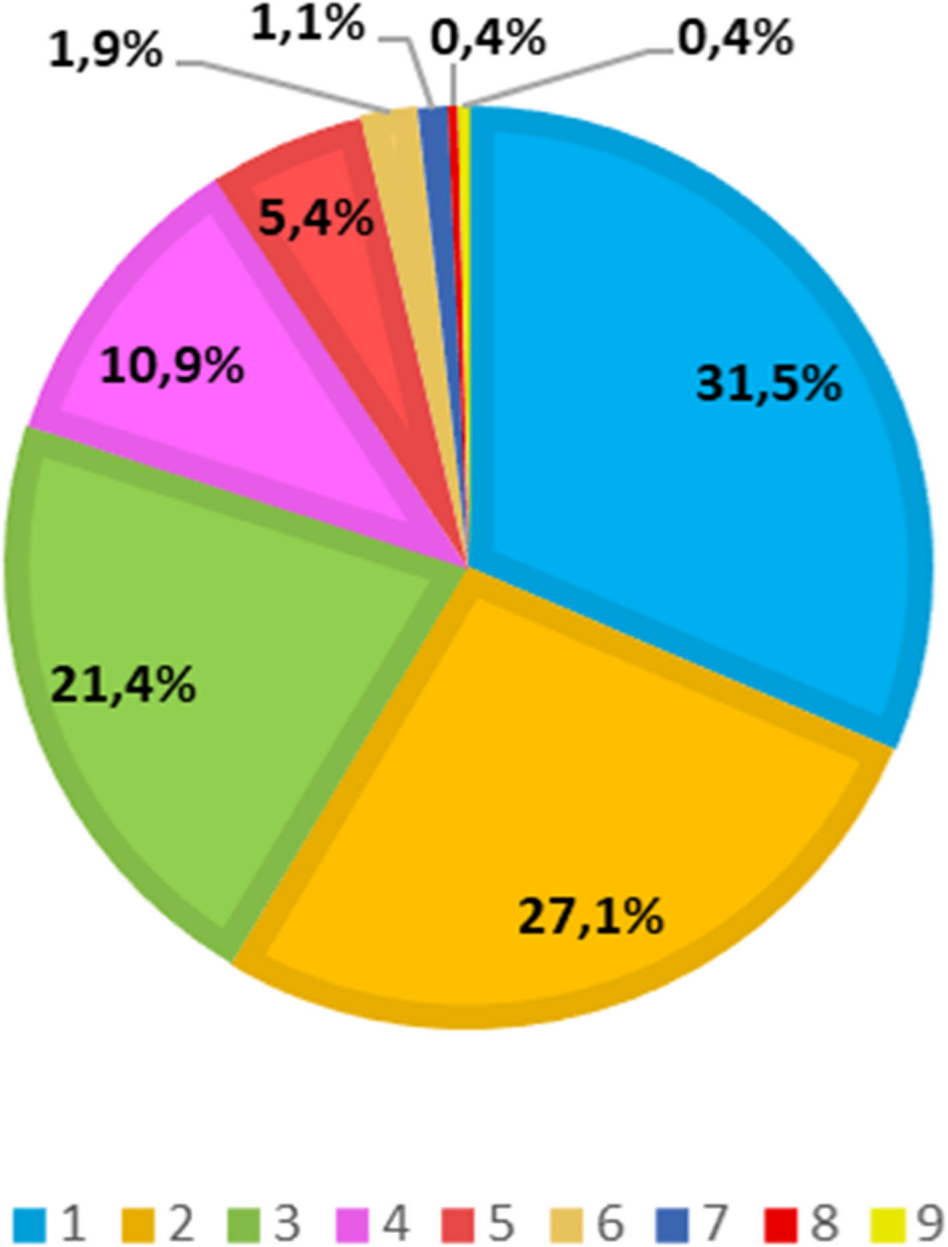
Number of diagnostic categories (ICD11) identified per participant (*n* = 572)

Regarding the number of identified diagnostic categories, most of the sample (27.1 %) had two identified categories, and the maximum number of identified diagnostic categories was nine (Fig. 2).

### Differences in functional and cognitive profiles, sociodemographic characteristics and multimorbidity

Pearson’s correlation was used to analyze the relationship between the participants’ age and average functional profile, and the higher the age, the worse the overall functional profile and the functional profile for all the ENCS domains, with a *p* < 0.05; the exception was the social relationship domain, for which no statistically significant differences were observed. The *t*-test revealed that participants with diseases of the nervous system presented worse results for the overall functional profile and in all ENCS domains, as well as on the MMSE (*p* < 0.001). Those with endocrine and/or metabolic diseases had worse results for the overall functional profile and in the domains of self-care and learning and mental functions (*p* < 0.05). There were no statistically significant differences for the other pathologies.

Pearson’s correlation for sociodemographic characteristics and the number of diseases was also analyzed, and the results indicated that older adults living in residential homes had more diseases than those who attended the day center (*p* < 0.05).

Table 3 shows the differences in the ENCS and MMSE scores according to the variables sex, marital status, education level, support level and multimorbidity.

**Table 3:** Differences in ENCS and MMSE scores according to sociodemographic characteristics and diagnostic categories

		MMSE		Functional profile	
*Variables*	*n*	Mean	*t*	Mean	*t*
*Sex:*			2.63**		-3.15**
Female	425	16.40		2.43	
Male	188	18.61		2.14	
*Spouse*			2.30*		-1.23
Yes	109	18.85		2.22	
No	504	16.70		2.37	
*Attended school*			-7.25***		4.40***
Yes	369	19.28		2.17	
No	244	13.75		2.60	
*Level of care*			6.40***		- 10.59***
Residential home	507	16.20		2.48	
Day center	106	21.27		1.67	
*Multimorbidity*					
Yes	390	16.74	1.52	2.47	- 4.13***
No	182	17.99		2.08	

***p<0.001

**p<0.01

*p<0.05

## Discussion

This study aimed to analyze the sociodemographic and pathological characteristics of elderly adults attending institutions for older adults and to evaluate the association between these variables and functioning and cognition.

The most notable finding was the high percentage of women in our sample (69.6 %). We know that women have greater longevity than men, a phenomenon known as the “feminization of aging” [21, 22]. However, in our sample, the number of women was more than twice the number of men, which is not consistent with data for women in the same age group living in Portugal (58.2 %). A study conducted in Portugal with older adults from the general population had a percentage of women of 52.9 % [23]. Another Portuguese study with 351 older people living at home had a percentage of women of 53.6 % [24]. These results indicate that a disproportionate number of women live in residential homes and day centers. In addition, women had a worse functional profile and more cognitive impairment than men, which indicates greater functional dependence. These data are in line with other international studies that indicate that women have higher rates of functional dependence [4, 25, 26] and cognitive deficits [26].

Regarding age, the older the age group was, the higher the proportion of the sample it comprised. Additionally, the mean age in this study (85.73 years) was higher than that in a Portuguese study conducted with older adults in the general population (80.16 years) [23]. This finding is to be expected given the need for increased care with increasing age and the need to resort to care services. Moreover, the mean age in this study was also higher than that in other international studies with older adults [27–29]. In fact, Portugal is the third oldest country in the European Union [30].

In relation to marital status, more than half of the participants were widowed, and only 17.8 % were married. The results indicate that married participants had better cognitive levels than unmarried participants. These data are in line with a recent longitudinal study conducted by the National Health and Aging Trends Study that concluded that unmarried older adults are particularly vulnerable to cognitive impairment, and marital status is a potentially important social protective factor [31]. Another study conducted in China with a sample of 2,498 older adults (> 55 years) concluded that being single or a widower was associated with higher cognitive impairments than being married, but only for men [32]. Another recent study concluded that social relationships can influence health and longevity, and their absence is a risk factor for premature death [33]. These data lead us to believe that the marital status of married individuals is a factor related to continuing to live at home. Thus, affective relationships should be given importance, and preventive measures to avoid cognitive impairment should be planned in advance and implemented when a spouse dies.

Most of the sample had attended school, but a significant percentage did not know how to read or write (31.8 %). Other studies with older adults showed similar data[34, 35]. Participants who had not attended school had a worse functional profile and more cognitive impairment than those who had attended school. Similar data were reported in a study of the Brazilian population in which illiterate individuals were the most dependent for instrumental activities of daily living [36]. Other studies concluded that a higher level of education is a predictor of better cognitive function [37, 38]. In addition, the higher an individual’s education level is, the better his or her physical and mental health [2, 39–41]. Considering these results, it is important to promote lifelong learning as a factor that contributes to healthy aging [42]. Thus, care models should focus on the promotion of literacy.

The participants who attended the day center and resided in their homes had better functional profiles and cognitive levels than those who resided in a residential center. These results are in line with those of other studies that indicate that functional dependence and cognitive impairment are predictive factors of institutionalization [43, 44]. In addition, factors such as living alone, not participating in recreational and social activities, not receiving visits from family and friends and a lack of social support are strong predictors of institutionalization in older adults [45]. Another important finding of our study is that the number of diseases was higher in the group of older adults living in residential centers. Multimorbidity may be another predictor of institutionalization, given its effect on the functional profile and, as a consequence, on the degree of dependence.

More than two-thirds of the sample had multimorbidity (68.2 %). Similar data were found in a study conducted in Scotland with older adults over 65 years of age in the general population [46]. Older adults with multimorbidity had a worse functional profile, and this finding was is in line with those of other studies with older adults [23, 47]. Participants with diseases of the nervous system, including dementias, had a worse functional profile and greater cognitive impairment and thus more dependence. These data are in line with those of other studies [48]. Scientific evidence shows that dementia is the main cause of disability in older adults [49]. Participants with endocrine, nutritional and metabolic diseases, including diabetes, had a worse functional profile. These results are in agreement with a study that indicated that Alzheimer’s disease and diabetes are among the diseases that contribute the most to disability [50].

### Limitations

The limitations of the present study include the fact that it is a cross-sectional study that does not allow the establishment of a cause-effect relationship between the variables. Another limitation is the fact that multimorbidity was defined according to the medical diagnostic categories of the ICD-10 rather than the pathology itself. These limitations must be considered in the analysis of the results.

## Conclusions

Understanding the factors that influence functional limitations, including cognitive limitations and multimorbidity, is an important step for the development of new care models for older adults in social support institutions. This study concluded that women, those who were illiterate, those who were institutionalized and older adults who had diseases of the nervous system had a worse functional profile and greater cognitive impairment. Those with multimorbidity had a worse functional profile, and those without a spouse had greater cognitive impairment.

Given the sociodemographic characteristics, functional and cognitive profiles and multimorbidity of older adults who attend institutions for older adults, it is necessary to adopt practices that focus on self-care. Considering the high multimorbidity and functional profile of older adults in these institutions, the social and health sectors must offer coordinated responses. In addition, care should be provided by health professionals qualified to provide assistance in self-care management, such as nurses, which is not the case in Portugal.

Therefore, it is necessary to develop new policies for implementing health promotion and disease prevention strategies in older adults in these institutions that promote active and healthy aging, as recommended by the WHO. Such efforts essentially involve the allocation of human resources in the area of health; the proper evaluation of functioning, including cognitive evaluation, on a regular basis to understand its evolution and to determine the effectiveness of care; and the adoption of care models focused on self-care management and the promotion of literacy and social relationships.

Participation in the study was explained to the participants, and informed consent was obtained from the participant or a legal representative if the participant was not able to provide it.

## Data Availability

The datasets used and/or analyzed during the current study are available from the corresponding author upon reasonable request.
